# Ab initio study of the vibrational spectra of amorphous boron nitride

**DOI:** 10.1038/s41598-024-56010-8

**Published:** 2024-04-04

**Authors:** David Hinojosa-Romero, Alexander Valladares, Renela M. Valladares, Isaías Rodríguez, Ariel A. Valladares

**Affiliations:** 1 Instituto de Investigaciones en Materiales, Universidad Nacional Autónoma de México, Apartado Postal 70-360, Ciudad Universitaria, CDMX, 04510 México; 2 Facultad de Ciencias, Universidad Nacional Autónoma de México, Apartado Postal 70-542, Ciudad Universitaria, CDMX, 04510 México

**Keywords:** Structure of solids and liquids, Condensed-matter physics, Theory and computation

## Abstract

Boron Nitride (BN) is an interesting polymorphic insulator that is commonly found in four different crystalline structures, each one with different electrical and mechanical properties which makes it an attractive material for technological and industrial applications. Seeking to improve its features, several experimental and simulational works have studied the amorphous phase (a-BN) focusing on electronic and structural properties, pressure-induced phase transformations, and a hydrogenated form of a-BN. By means of ab initio Molecular Dynamics and our well-proven amorphization process known as the *undermelt-quench* approach, herein three amorphous supercells were computationally generated, two with 216 atoms (densities of 2.04 and 2.80 g cm^−3^) and a third one with 254 atoms (density of 3.48 g cm^−3^). The topology, the vibrational density of states and some thermodynamic properties of the three samples are reported and compared with existing experiments and with other computational results.

## Introduction

Boron is an atom with the electronic structure dictated by a He-like core, plus 2 electrons in the *2s* shell and 1 electron in the *2p* shell; atomic nitrogen, located on the same row of the Periodic Table, has also a He-like core plus 2 *2s* and 3 *2p* electrons. When alloyed in equal quantities in the solid state, BN, four crystalline structures may appear, two three-dimensional (3-d) high-density structures and two two-dimensional (2-d) low-density structures^1^. In the bulk, one has the 3-d forms: cubic BN (*c*-BN), which has the zinc blende structure and another structure, rare hexagonal, the wurtzite (*w*-BN)^2^. *c*-BN is a very hard material^3^ since it is tetrahedral and covalently bonded; *w*-BN is also covalently bonded in a hexagonal arrangement. In 2-d one has the layered hexagonal BN (*h*-BN) and the rhombohedral BN (*r*-BN) structures. The *h*-BN structure consists of alternating B and N atoms in layers, covalently bonded and its name is due to the hexagonal rings formed by the elements. *r*-BN consists of three layers arranged in a stacking structure. In both cases the van der Waals forces are responsible for maintaining the layers together^4^.

Being isoelectronic to carbon, c-BN and h-BN are expected to have physical properties analogous to diamond and graphite, respectively, this is why earlier phase diagrams for BN^5^ reported h-BN as the stable phase at ambient temperature and pressure conditions. However, later investigations^6–8^ reported c-BN as the stable phase. This dilemma, which has prevailed over the years, has not stopped the extensive experimental and theoretical research for these two most common modifications of BN since it is a promising material for industrial applications^9–18^.

On the other hand, the amorphous phase of BN (a-BN) has been studied less than its crystalline phase^19–28^ and therefore correlation functions for the amorphous are scarce. One of the reasons for this deficit in knowledge may be due to the difficulty to study, both computationally and experimentally, the amorphous phase. In general, computationally generating amorphous covalently-bonded materials is a challenge. Solids like carbon reach different final structures and different bonding depending on the density of the starting material^29^. Also, the fact that the melting temperatures of this covalent solids are in the thousands of kelvins and the fact that some methods liquefy the specimens first and then quench it from the liquid, the so-called *melt-and-quench* procedure involves the consideration of very high temperatures and thus the possible appearance of liquid characteristics in the final structures. The theoretical approach could be quantum mechanical or classical^30^ and even though with classical potentials it is possible to deal with thousands of atoms in a supercell, the description is not as adequate as the quantum mechanical one that better represents the nature of the chemical bond.

Specifically for BN, the first experimental attempts recorded to obtain amorphous samples are by Rand and Roberts^19^ and Hirayama and Shohno^20^, who produced samples of “clear, vitreous films”^21^ reminiscent of disordered materials. A recent paper^27^ analyze the possible use of amorphous BN as ultralow dielectric-constant material which may be relevant for some technological applications.

Moreover, although the thermal properties due to the disordered lattices are important to understand the behavior of the material, to the authors’ best knowledge not much has been done along these lines for BN. In general, it is well established that for periodic solids at low temperatures, the Debye approximation, a non-atomistic continuous approach, may be used to describe the behavior of the vibrations. This implies that the vibrational Density of States (vDoS, or *F(ω*)) for low *ω* should vary as *ω*^*2*^ and the specific heat at constant volume should vary as *T*^*3*^*.* One would expect that if this happens for crystalline materials at low temperatures it should also happen for the “homogeneous” structure of the amorphous counterparts. However, experimentalist found that the Debye approximation does not work too well in the amorphous phases^31^ and that a high percentage of low-frequency phonon modes appear together with the decreasing and widening of the crystalline optical peaks. In 2006, these experimental results led us to study the phonon density of states in an amorphous structure of pure silicon, and the findings corroborated the observation given above^32^.

Concerning the vDoS of BN, Wu and Han^28^ performed a calculation of a defective-to-amorphous hexagonal boron nitride monolayer and they obtained some vDoS customizing the nature of the defects, studying their relevance to the thermal conductivity. The classical empirical Tersoff potential was used in their work as an approximation to the quantum nature of the interatomic interactions.

After the limited number of related works, the reason for the present study was to calculate the vibrational density of states of three (different density) amorphous samples of boron-nitrogen to investigate what happens with the crystalline vDoS after the amorphizing process. With this in mind, we proceeded to the creation of the amorphous samples and studied them by obtaining the Pair Distribution Functions (PDFs), the reduced PDFs (rPDFs), the partial PDFs (pPDFs), the Plane Angle Distribution Functions (PADs), and the vDoS. The amorphous samples were obtained by means of ab initio Molecular Dynamics (MD) and our *undermelt-quench* approach^33,34^, a method in which no melting of the material is involved and the quantum methods are used to better describe the electronic structure. Also, thermodynamic properties, such as the internal energy and the specific heat at constant volume, as a function of temperature, are also calculated and analyzed to provide some examples of the lattice-vibrations contributions to thermal properties of a-BN.

## Methodology

The simulation of amorphous semiconductors by means of MD is traditionally done following the *melt-and-quench* (M-Q) procedure in an attempt to mimic the experimental route to obtain such structures; however, it was shown that this method produces overcoordinated structures in the amorphous phase. Given the fact that liquid phases of some semiconductors are metallic with a nearest-neighbor number larger than four, we surmised that overcoordination appears because the samples are liquified first and then solidified. This conjecture led to the development of the *undermelt-quench* (U-Q) approach in which the maximum temperature reached during the MD process does not exceed the liquid temperature, thus preventing from obtaining final structures with possible liquid characteristics^33^.

In general, the *undermelt-quench* approach considers a starting unstable supercell for the system, usually with an atomic distribution that does not correspond to the crystalline phase of the material which helps the disordering process of the MD. The unstable structure is subjected to a heating ramp which takes the system, in 100 steps, from 300 K to a temperature below the liquidus temperature, followed by a cooling ramp that reaches 0 K as close as possible. The heating and cooling slopes are, in absolute value, the same. After the MD, a Geometry Optimization (GO) process for the atomic positions is carried out to allow the atoms accommodate in their local minimum energy positions. This method has proven to generate amorphous samples congruent with reality^34–37^.

Specifically for this work and considering the mass density to be the relevant factor in our simulations^29^ because of the diverse 2-d and 3-d atomic topologies that crystalline BN has, the MD and GO processes were done for three different supercells of densities 2.04, 2.80 and 3.48 g cm^−3^. The 2.04 g cm^−3^ density was chosen to be the same as the theoretical model of Durandurdu^25^, in accordance with the experimental range of 1.7–2.1 g cm^–3^^21^. The 3.48 g cm^−3^ density was chosen to be the same as one of the samples reported in the experimental work of Kurdyumov, et al*.*^38^ [See also Ref.^39^]. The 2.80 g cm^−3^ density was chosen as the (rounded) average between the other two values, value that is between the two densities, 2.0 and 3.0 g cm^–3^, for the models studied by Mc Culloch et al.^24^.

As required by the U-Q method, two different starting unstable structures were used. For the densities of 2.04 g cm^−3^ and 2.80 g cm^−3^, 108 borons and 108 nitrogens randomly occupied the ordered positions of a tetrahedral, diamond-like structure, within two 216-atom supercells, one of them with an edge-length of 12.96 Å (for the low density sample) and the other with an edge-length of 11.67 Å (for the high density sample). For the supercell with a density of 3.48 g cm^−3^ a 256-atom, face-centered-cubic structure with an edge length of 11.48 Å was chosen as the unstable starting supercell, since the *c-BN* and *w-BN* systems crystallize in diamond-like and hexagonal closed-packed structures, respectively, both having an approximate density of 3.6 g cm^−3^^21^.

For the MD processes, the initial velocities assigned to each atom follow a Maxwell–Boltzmann distribution at 300 K; the subsequent temperature control is carried out within an NVT (constant Number of atoms, Volume, and Temperature) ensemble by means of the simple Nosé-Hoover thermostat^40,41^, with a 0.5 Q-ratio coupling parameter between the thermal bath and the system. Since the liquidus temperature of BN is above 3000 K at ambient pressure^42^, heating ramps for the U-Q processes reached a maximum temperature of approximately 2000 K in 100 steps, followed immediately by cooling ramps that reached approximately 5.5 K in 118 steps. The time step for the MD processes was 1.5 fs, resulting in a total simulation time of 327 fs. As a complement, the *melt-and-quench* method was also executed in order to spot relevant structural differences through the comparison between the obtained correlation functions; the same parameters were employed for the M-Q as for the U-Q method except that the highest temperature reached was approximately 3200 K. The GO processes were carried out for both methods using delocalized internal coordinates with the following convergence thresholds: 2.72 × 10^–4^ eV for energy, 5.44 × 10^–2^ eV Å^−1^ for maximum force, and 5 × 10^–3^ Å for maximum displacement. After the GO, cohesive energies (${|E}_{b}|$) were calculated as the absolute value of the binding energy (${E}_{b}$) at equilibrium^43^, which in turn is determined from: ${E}_{b}={E}_{SC}-\sum {E}_{A}$, where ${E}_{SC}$ is the total energy of the supercell and ${E}_{A}$ is the energy of each individual, isolated atom.

There are several ways to describe the amorphous structures of materials, among which the pair distribution functions (PDFs), total and partial, are very frequently reported. In an attempt to do a more complete description, an analysis of the distribution of rings, of plane angles, of tetrahedral angles, etc. should be included. However, experimentally most of these distributions are hardly reported and that is why we have only used PDFs in the present paper. Thus, once the amorphous structures for the three samples were obtained with both the U-Q and the M-Q methods, their structures were determined with the code **Correlation**^44^. The total, partial, and reduced PDFs were generated using a bin-width of 0.1 Å. For the PAD functions, a 1-degree bin-width was employed, and the criterion to consider two atoms as bonded was that their interatomic distances do not exceed 1.3 times the sum of their covalent radii (0.710 Å for boron and 0.740 Å for nitrogen). Subsequent smoothing with a 3-point Fast-Fourier-Transform (FFT) was applied to all data in order to have curves that simulate the bulk material and to compare with experiments and other simulations.

For the calculation of the vibrational eigenmodes, the mass-weighted Hessian matrices were computed numerically using finite differences of first energy derivatives with a step size of 0.005 Å. The vDoS were found by smoothing, with a 3-point FFT, the phonon energy distributions obtained by counting the number of eigenmodes of each supercell located within a 1.5 meV interval. The vDoS reported in this paper are normalized to 1 for two reasons: to make an adequate comparison between the three studied densities since the number of atoms within the supercells are different, and to correctly use *F(ω)* in the calculation of the vibrational contributions to the internal energy and the constant volume specific heat (see Eqs. (1) and (2) below, and Reference^45^).

Since the contribution of the lattice vibrations to the thermodynamic properties of materials as a function of temperature can be estimated through the calculated vDoS^46,47^, the internal energy, *ΔE*, and the constant-volume specific heat, *C*_*v*_, are also reported. The evaluations of both thermodynamic functions are done following the equations^46^:

1 $$\Delta E=3nN\frac{\hslash }{2}{\int }_{0}^{{\omega }_{L}}\omega {\text{coth}}\left(\frac{\hslash \omega }{2{k}_{B}T}\right)F\left(\omega \right)d\omega ,$$

2 $${C}_{v}=3nN{k}_{B}{\int }_{0}^{{\omega }_{L}}{\left(\frac{\hslash \omega }{2{k}_{B}T}\right)}^{2} {{\text{csch}}}^{2}\left(\frac{\hslash \omega }{2{k}_{B}T}\right)F\left(\omega \right)d\omega$$

where *n* is the number of atoms per unit cell (equal to 216 for the 2.04 and the 2.48 g cm^−3^ systems, and equal to 256 for the 3.48 g cm^−3^ system), *N* is the number of unit cells (equal to one since the supercell can be considered as the unit cell), *ω*_*L*_ is the largest phonon frequency, and *F(ω)* is the normalized vDoS, *T* is the temperature, *ħ* is Planck´s constant divided by 2*π*, and *k*_*B*_ is Boltzmann constant.

In order to compare *C*_*v*_* / T*^*3*^ among our structures, the specific heat was scaled with the Debye form of *C*_*v*_, in which the scaling factor *C*_*D*_ was defined as:

3 $${C}_{V}\approx \frac{12{\pi }^{4}n{k}_{B}}{5{T}_{D}^{3}}{T}^{3}={C}_{D}{T}^{3}$$

where *T*_*D*_ is the Debye temperature of each sample, calculated from ^48^:

4 $${T}_{D}= \frac{\hslash }{{k}_{B}}{\text{exp}}\left(\frac{1}{3}+\frac{{\int }_{0}^{{\omega }_{L}}ln\left(\omega \right)F\left(\omega \right)d\omega }{{\int }_{0}^{{\omega }_{L}}F\left(\omega \right)d\omega }\right)$$

Similarly, the comparison among our calculated vDoS was done by the scaling factor *F*_*D*_ defined as:

5 $$F\left(\omega \right)\approx \frac{3n{\hslash }^{3}}{2{\pi }^{2}{k}_{B}^{3}{T}_{D}^{3}}{\omega }^{2}= {F}_{D}{\omega }^{2}$$

All the simulations were spin-unrestricted, all-electron calculations done within the DFT framework as implemented in the DMol^3^ code^49^ included in the Materials Studio software^50^. Atomic orbitals for boron and nitrogen are described by a Double Numerical plus polarization d-function basis (DND) with an orbital cutoff of 4.1 Å. The exchange–correlation functional was treated under the Local Density Approximation (LDA) parameterized by Vosko, Wilk and Nusair (VWN)^51^. The Self-Consistent-Field density convergence threshold was set to 1 × 10^–6^ and all the numerical integrations are done at the *Γ*-point with a fine integration grid (as defined by DMol^3^ ^49^).

## Results and discussion

Since we are dealing with amorphous structures of BN, it should be kept in mind that the atomic configurations reached are local energy minimum ones an one should not expect to find the *minimum minimorum* of the energies for each structure; thus, the cohesive energy (${|E}_{b}|$) is provided next as one of the proofs of obtaining amorphous structures from our simulations when compared with the crystalline values. It was found that, accordingly for the 2.04, 2.80, and 3.48 g cm^−3^ densities, ${|E}_{b}|$ increases up to 6.784, 6.776, and 6.993 eV per atom for the structures generated by the U-Q method, and up to 5.979, 5.944, and 6.074 eV per atom for the ones generated by the M-Q method. Those values must be compared with those reported for the crystalline structures which are of the order of 8 eV per atom^52,53^. Apart from the fact that the calculated values for the binding energies should be taken with caution because of the known overestimation of binding energies in the LDA approximation^46^, the U-Q method systematically produced structures which are more stable than the ones generated by the M-Q for the three samples. Thus, the properties of the a-BN analyzed in this paper will correspond to the structures generated by the U-Q method.

### Correlation functions

**Figure 1:**
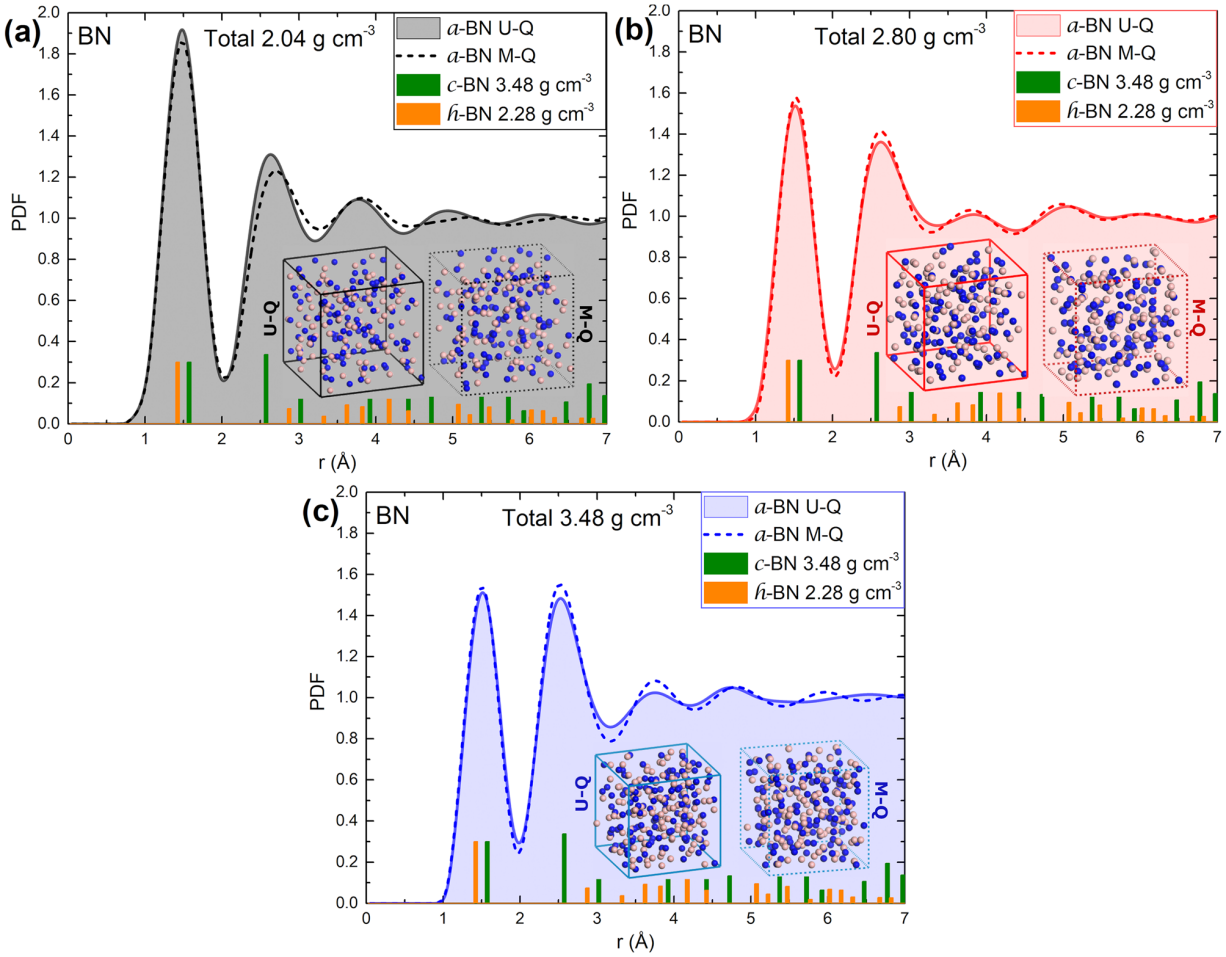
Total PDFs for our *undermelt-quench* (U-Q, shaded curves) and the *melt-and-quench* (M-Q, dashed curves) methods corresponding to the samples of density (**a**) 2.04 g cm^−3^ (black), (**b**) 2.80 g cm^−3^ (red), and (**c**) 3.48 g cm^−3^ (blue). The PDFs for the c-BN (green) and the h-BN (orange) crystalline structures are shown for comparison. Final supercells for the U-Q (solid lattice lines) and the M-Q (broken lattice lines) methods are shown in the insets.

Figure 1 represent total PDFs for our *undermelt-quench* (U-Q, shaded curves) and the *melt-and-quench* (M-Q, dashed curves) methods, corresponding to the samples of density (a) 2.04 g cm^−3^ (black), (b) 2.80 g cm^−3^ (red), and (c) 3.48 g cm^−3^ (blue). Also, the PDFs for the c-BN (green) and the h-BN (orange) crystalline structures are shown for comparison. The atomic topologies of the final supercells for the U-Q (solid lattice lines) and the M-Q (broken lattice lines) methods are also shown in the insets, where boron is represented by spheres with a light color, and nitrogen by spheres with a dark color. The atomic structures of the supercells obtained by the two methods are very different; however, the PDFs are very similar and difficult to tell them apart. Nevertheless, as mentioned before, the generated structures from the U-Q method are more stable than those from the M-Q approach.

**Figure 2:**
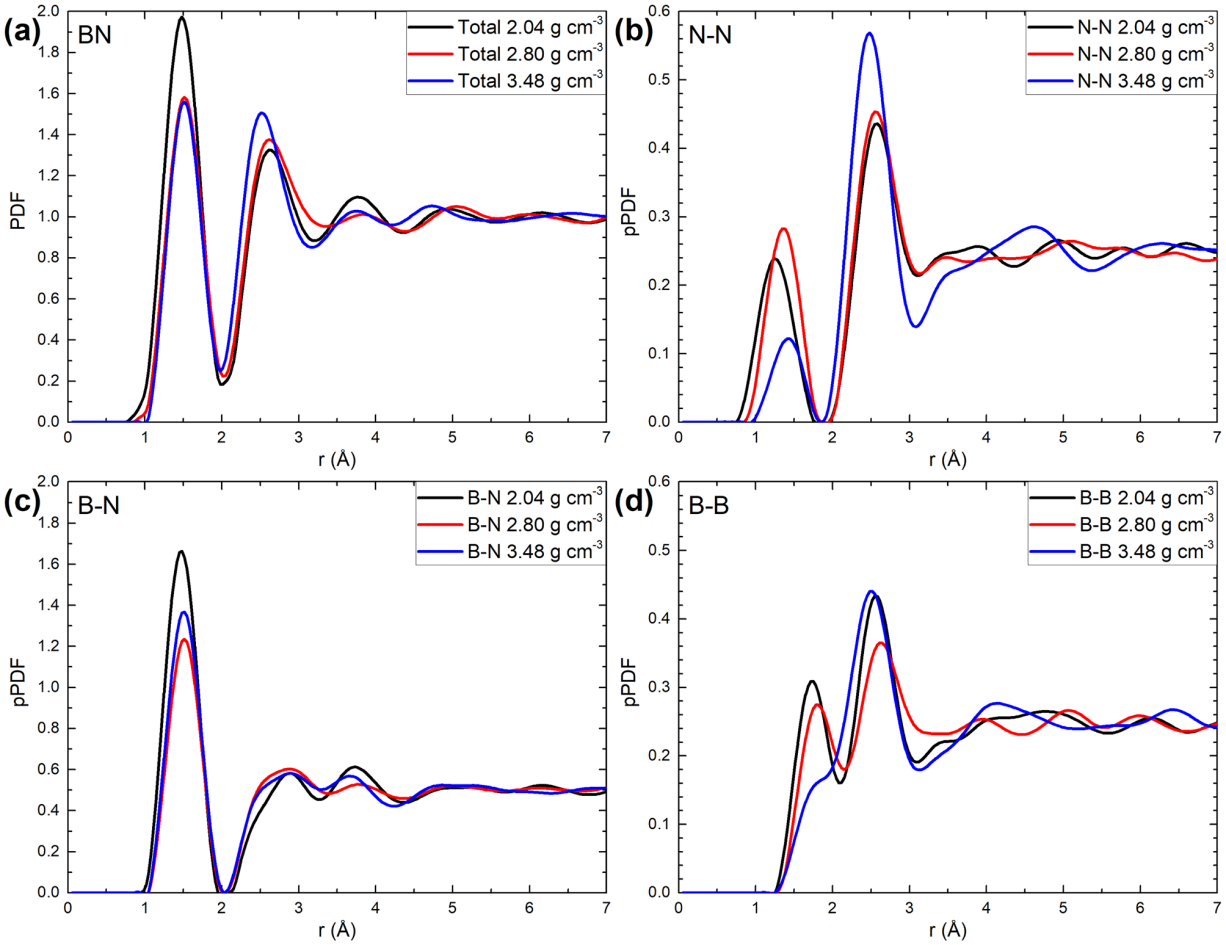
(**a**) Total PDFs. Weighted partials for (**b**) N–N, (**c**) B-N, and (**d**) B-B for all densities. Black lines correspond to the density of 2.04 g cm^−3^; red lines, to the density of 2.80 g cm^−3^; blue lines, to the density of 3.48 g cm^−3^.

In Fig. 2 the PDFs, total and partial, for the three concentrations are depicted. As shown in Fig. 2a and Table 1, the positions for the first two peaks of the total PDF for the 2.80, and 3.48 g cm^−3^ densities are the same: 1.55 Å, whereas for the 2.04 g cm^−3^ density, the peak is located at the smaller position of 1.45 Å. Given the fact that the amorphization process tends to maintain constant the nearest-neighbors interatomic distances, the decrement in the value of the first peak position for the lowest-density sample is an indication of a subjacent structural difference between our amorphous samples. In particular, an incipient porosity may affect the interatomic distances at the surface of the pores. Indeed, a Connolly surface analysis^54^ with a probe of radius of 1.0 Å gives an approximate value of 4% of free volume for the 2.04 g cm^−3^ sample, contrasting with the 0% of free volume for the other two densities. The effect of porosity from the partial PDFs is also evident for the first-peak positions in the B-N subset, shown in Fig. 2c and Table 1. Although less noticeable due to the smaller first-peak height, porosity is also manifested in the N–N and B-B subsets, shown respectively in Fig. 2b,d. The differences in the positions and the heights for the first peaks of N–N, B-N, and B-B (see Table 1) may be responsible for the difference in the high-frequency modes observed for the amorphous samples as will be shown below.

**Table 1:** Positions for the first two peaks of the PDFs and pPDF for all samples.

Peak (Å)		1st peak			2nd peak		
Density (g cm^−3^)		2.04	2.80	3.48	2.04	2.80	3.48
PDF total		1.45	1.55	1.55	2.65	2.65	2.55
pPDF	N–N	1.25	1.35	1.45	2.55	2.55	2.45
	B-N	1.45	1.55	1.55	2.85	2.85	2.85
	B-B	1.75	1.85	1.85*	2.55	2.65	2.55

*The first peak position of the 3.48 g cm^−3^ sample was not clearly identifiable and the peak finder tool from Origin software was employed to report this value.

It is noticeable that the number of alike nitrogen atoms within the first coordination shell, (quantity that is proportional to the height and width of the first peak in the N–N pPDF (Fig. 2b), diminishes for the system with the density of 3.48 g cm^−3^, whereas it remains almost constant for the other two densities. This behavior is more evident in the B-B pPDF (Fig. 2d) in which the position of the first peak for the 3.48 g cm^−3^ is not clearly defined as the other two since it becomes a shoulder for the second peak. This structural information requires an extended analysis beyond the scope of this work since it is necessary to study the electronic structure in detail for the three samples in order to understand this behavior.

**Figure 3:**
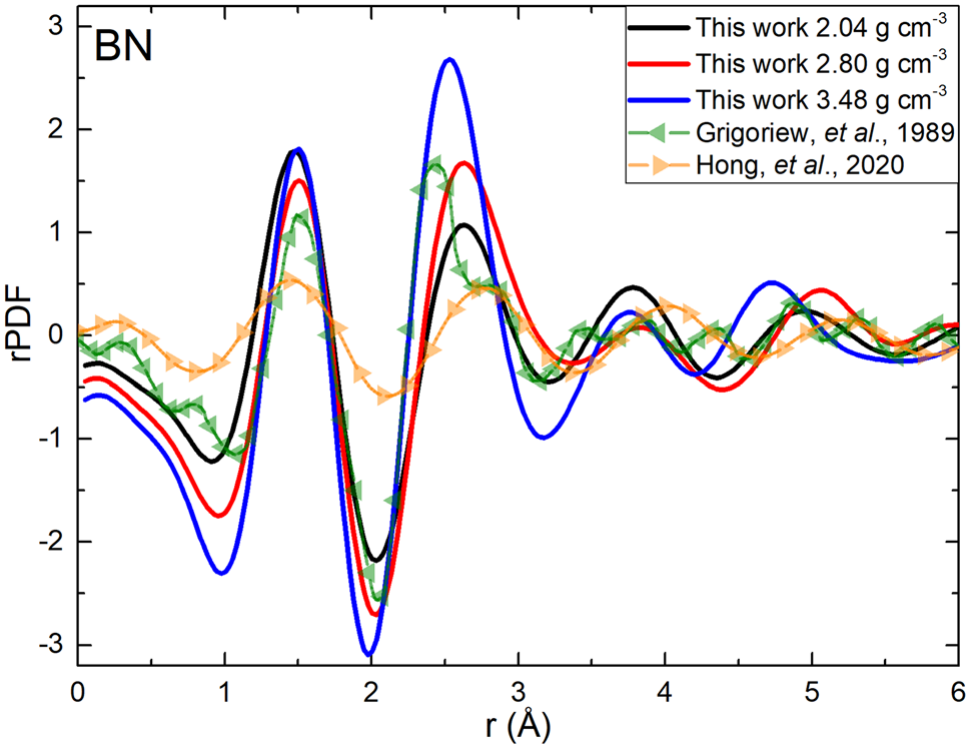
Total reduced Pair Distribution Functions for our three specimens and the two experimental results reported in the literature^27,55^.

Figure 3 represents a comparison of our rPDFs, G(r), and those reported by experimentalists^27,55^. The discrepancies are notable. It was expected not to find agreement with the structural measurements of the pioneering work by Grigoriew et al.^55^, since the existence of “admixtures of crystalline phases” in some of their thin-film samples is reported. From the very recent work of Hong et al*.*^27^ (density between 2.1 and 2.3 g cm^−3^), it is surprising that they only reported the rPDF from their electron diffraction data and not from their classical molecular dynamics simulation; however, their report of their first-peak position at 1.44 Å agrees very well with our 1.45 Å value. The reasons for the lack of agreement with Hong et al*.* results’ are multiple, primarily the missing information on the parameters they used to obtain their rPDF.

**Figure 4:**
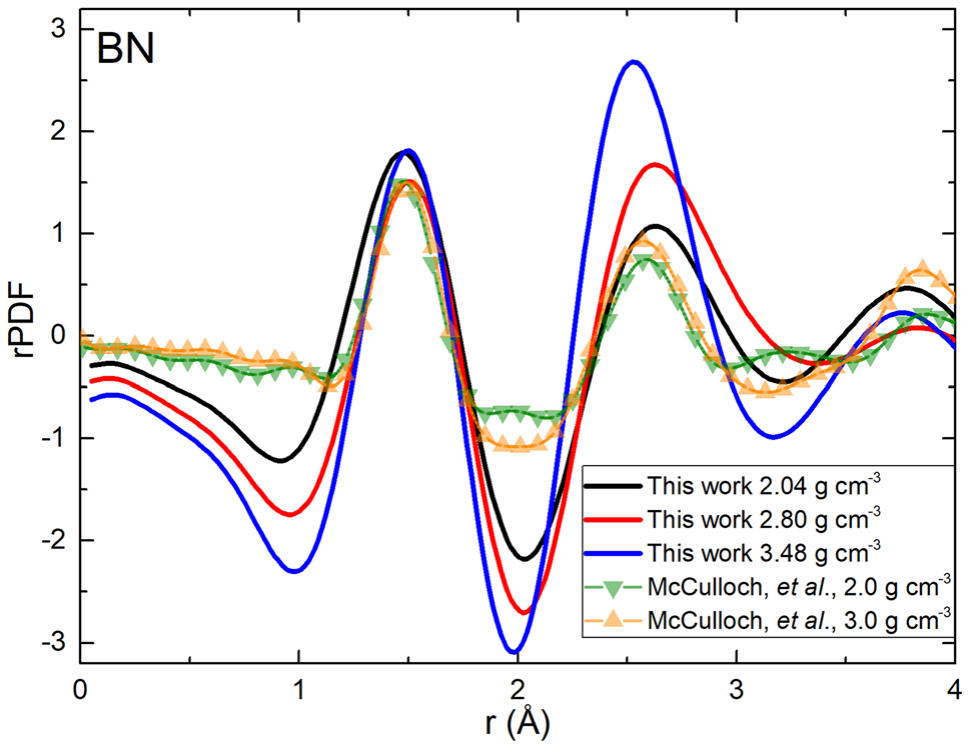
Comparison of the rPDFs obtained by computer simulations for samples very close in densities, 2.0 and 3.0 g cm^−3^ of McCulloch et al*.* and our work: 2.04, 2.80 and 3.48 g cm^−3^ ^24^.

In 2000, McCulloch and collaborators^24^ applied the Car-Parrinello molecular dynamics approach to two boron-nitrogen supercells (with densities of 2.0 and 3.0 g cm^−3^). To amorphize the material they liquefied it and quenched it to solid temperatures. As discussed before, doing this process may induce the appearance of some liquid-like characteristics in the resulting amorphous structures. Also, they used 64 atoms in the simulations, as opposed to the supercells containing of the order of 200 atoms used in this work. Even more, their calculations employed pseudopotentials, contrary to the all-electron calculations from this work. These three factors may explain the subtle differences between our results and McCulloch´s, presented in Fig. 4, given the fact that in both works the density functional approach was applied.

**Figure 5:**
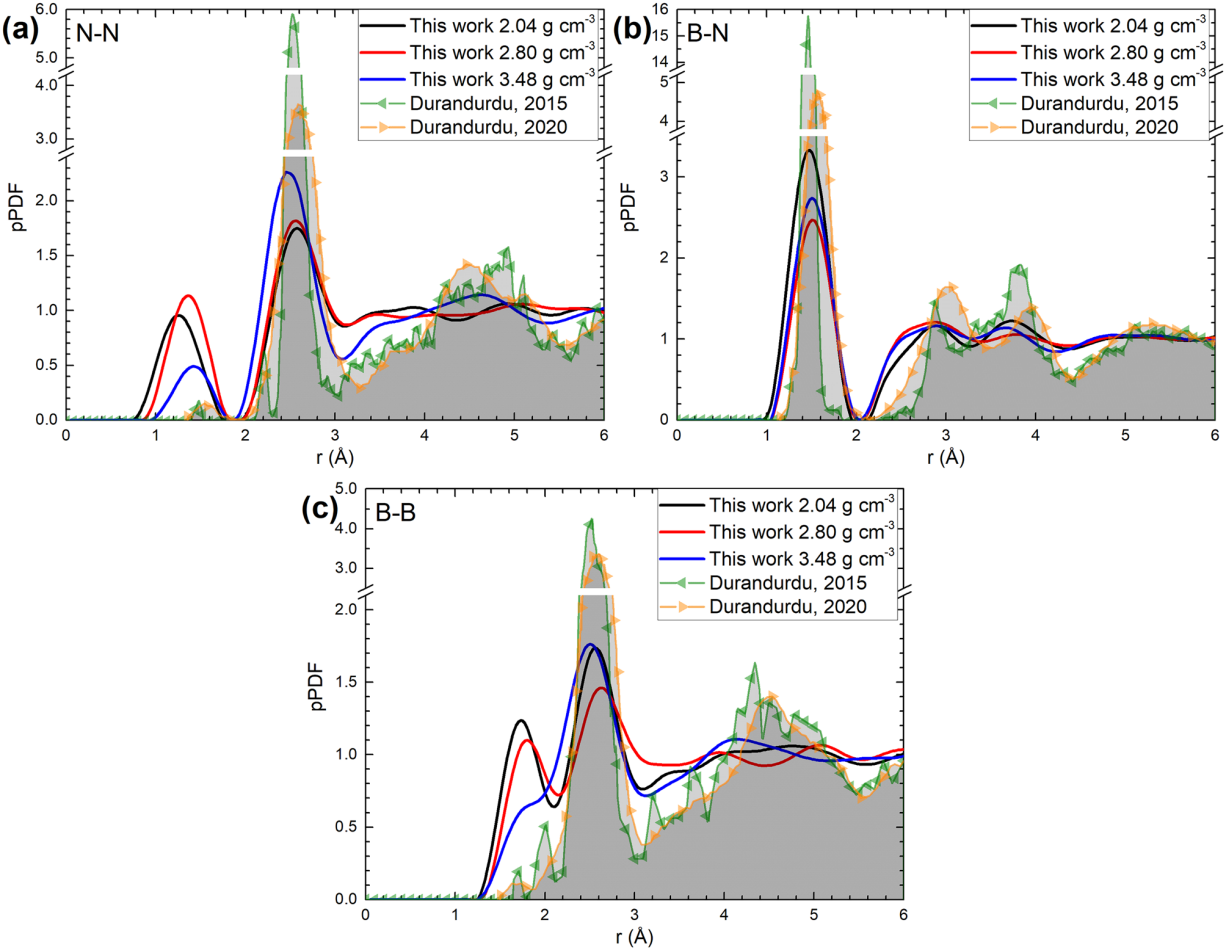
Comparison of Durandurdu´s results^25,26^ with ours for the PDF partials (**a**) N–N, (**b**) B-N, and (**c**) B-B.

**Figure 6:**
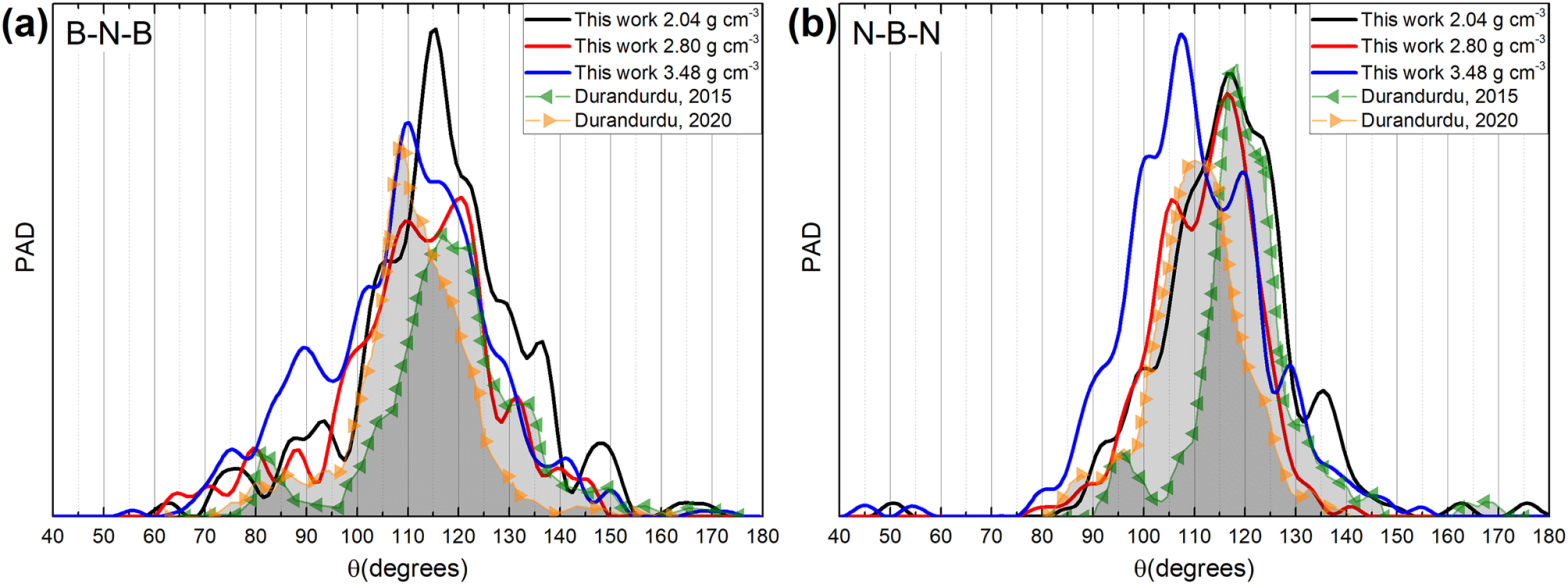
Comparison of Durandurdu´s results ^25,26^ with ours for the partial PADs (**a**) B-N-B and (**b**) N-B-N.

Simulational results by Durandurdu are also included here for comparison^25,26^; the discrepancies are evident. The reason being that the way in which he prepared the amorphous samples is very different from ours since the starting structures are different and so are the maximum temperatures reached during the MD processes; this may respond to different conceptions of the amorphous solids he wanted to generate and Fig. 5 for the partial PDFs makes this evident. This is also the case for the PADs shown in Fig. 6. Due to the fact that they reported the pPDFs and the PADs, it was possible to compare his findings with ours. The plane angle distribution functions, Fig. 6, give idea of the average structures by analyzing the prominent angles that appear in the PADs and, by extension, this also gives information about the state of hybridization the constituents have in the amorphous form.

### The vibrational density of states

**Figure 7:**
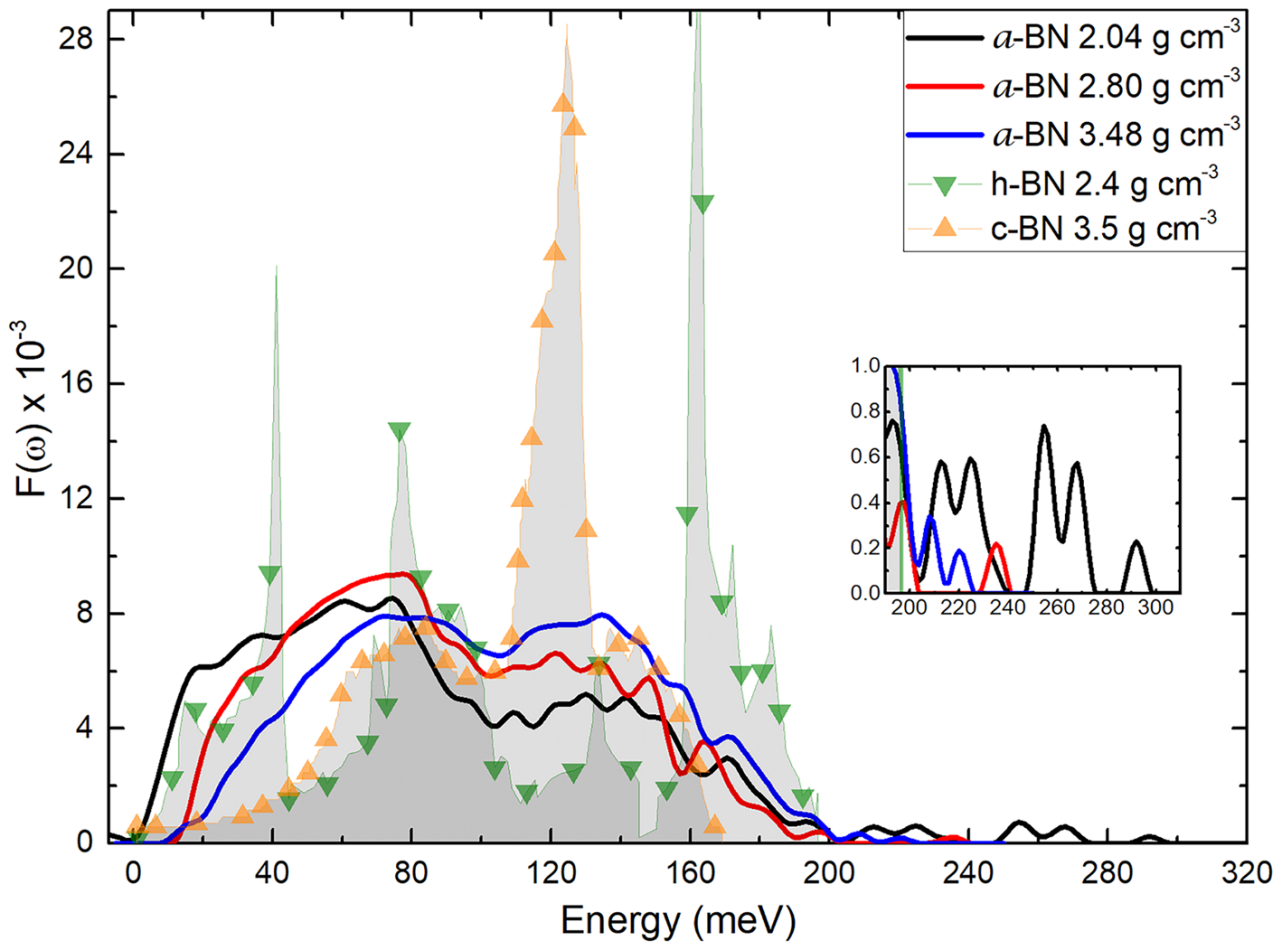
Vibrational densities of states for the three amorphous samples studied in this work compared to the results for the crystalline calculated hexagonal ( )^39^ and the crystalline experimental cubic ( )^56^ structures. The inset shows the vibrational modes for the interval (200–300) meV.

In Fig. 7, the calculated vDoS for our three samples is displayed; it is seen that the three develop a larger concentration of low-frequency modes, soft-phonon modes, and the high frequency modes almost disappear. Those low-frequency modes are located around 80 meV with a timid attempt to rescue some of the high frequency modes of the material at about 140 meV. The lowest density sample displays a greater percentage of high frequency modes compared to the highest density one; the same phenomenon appears for *ω* ~ *0*, which implies that the less dense sample has more low frequency modes than the high-density samples. This may be due to the fact that in the amorphous low-density sample the interatomic distances may be larger for some atomic pairs with the consequent decrease of the oscillator force, thereby diminishing the frequency of the associated modes, as a result of the incipient porosity of the material at this density. Even more, the frequency modes above 240 meV, present for the lowest density sample but absent for the other two densities (see inset of Fig. 7), are attributable to the difference in the interatomic distances (see Table 1), shorter for N–N, B–N, and B–B in the 2.04 g cm^−3^ sample compared to the other two supercells, due to a possible tendency to clustering of similar pair of atoms (molecule-forming) thereby decreasing their interatomic distances and consequently increasing the vibrational frequencies of similar atom pairs. The modes of the amorphous samples for other densities should be investigated to confirm this conjecture.

### Some thermodynamics

**Figure 8:**
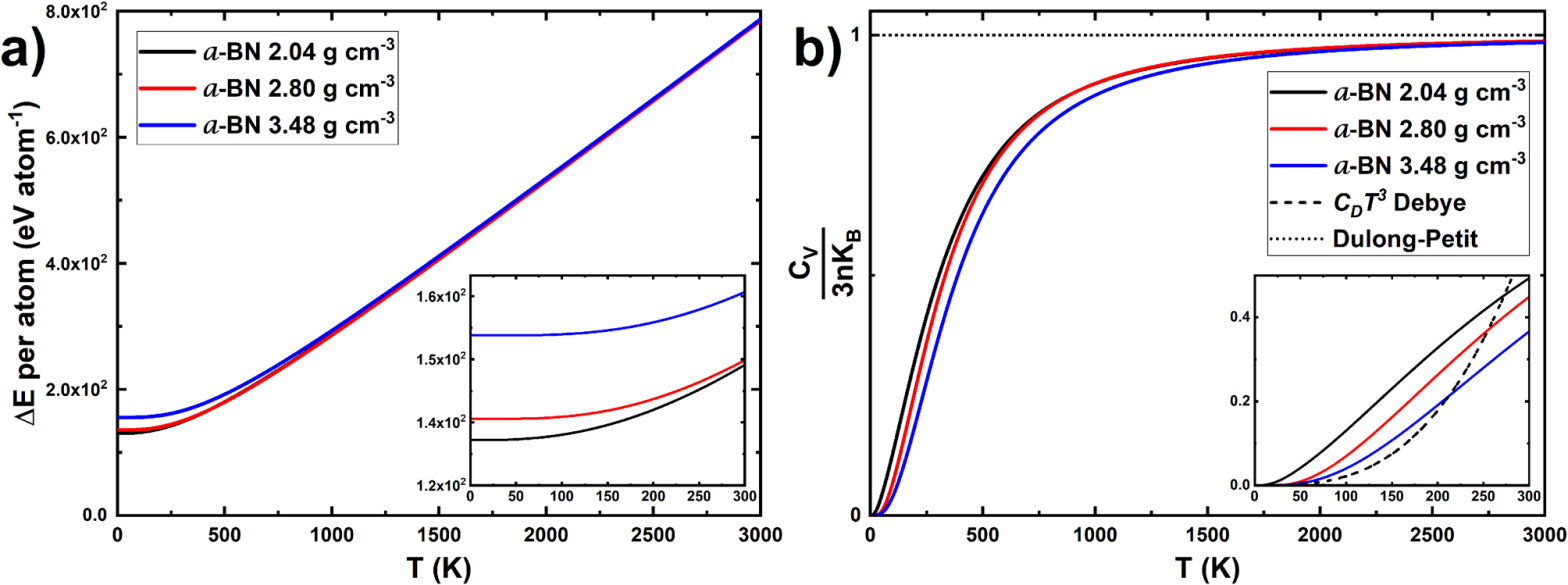
(**a**) Calculated internal energies per atom for the three samples studied. The inset shows the (0–300) K interval. (**b**) Constant volume specific heats scaled by the Dulong-Petit law, *3nk*_*B*_, which is shown as the constant horizontal dotted line. The interval (0–300) K is also displayed in the inset where the *T*^3^ Debye behavior for crystalline materials is shown as the dashed curve.

In Fig. 8, the behavior for the internal energy and specific heat is observed. Because of the extensive nature of these properties (see Eqs. (1) and (2)), an intensive alternative was used in order to compare the three samples: the internal energy, Fig. 8a, was divided by the number of atoms, *n*, in each cell (216 atoms for 2.04 g cm^−3^ and 2.80 g cm^−3^, and 256 atoms for 3.48 g cm^−3^); and the specific heat, Fig. 8b, was divided by the Dulong-Petit law (*3nk*_*B*_). As it is shown in the inset of Fig. 8b, the *C*_*V*_ behavior of our amorphous samples, at low-intermediate temperatures, does not follow the Debye *T*^3^ law.

**Figure 9:**
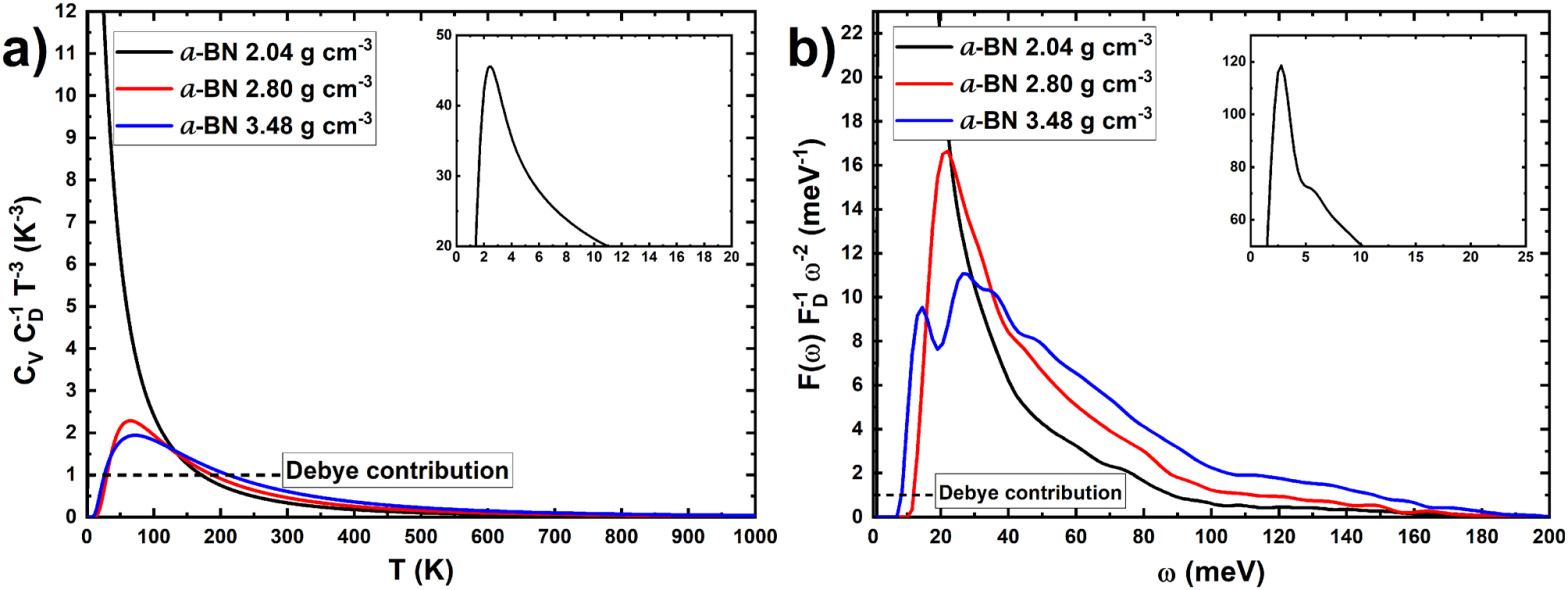
(**a**) *C*_*v*_* /T*^*3*^ (= *C*_*D*_) scaled by the *T*^*3*^ Debye dependence for the specific heat. (**b**) *F(ħω)/(ħω)*^2^ (= *F*_*D*_* /ħ*^2^) scaled by *(ħω)*^2^, the Debye contribution to the vDoS, for the three samples. The Debye contributions are shown as the dashed black lines. The insets display the corresponding maximum for the 2.04 g cm^−3^ sample.

As a further proof of the amorphous character of our samples and to make evident the deviations with the crystalline-solid expectations, the *C*_*v*_ and *F(ω)* are scaled, and the results explained in Fig. 9.

When the specific heat is divided by *T*^3^ and plotted as a function of temperature, Fig. 9a (see also Eq. (3) with *T*_*D*_ equaling 1129 K for the 2.04 g cm^−3^, 1294 K for the 2.80 g cm^−3^, and 1518 K for the 3.48 g cm^−3^ samples and calculated from Eq. 4), a straight horizontal line is expected at low temperatures for crystalline materials. In our case for the amorphous structures, a bump is observed at 2.5 K for the 2.04 g cm^−3^ sample (inset of Fig. 9a), at around 64.8 K for the 2.80 g cm^−3^ sample, and at around 72.1 K for the 3.48 g cm^−3^ sample. These maxima seem to be of the same order of magnitude of other amorphous borates like the ones reported by Kojima and Kawaji^57^ which ranged from 6 K for lithium borate to around 15 K for cesium borate.

When the vDoS is divided by *ω*^*2*^ (see Eq. (5) with the same Debye temperatures reported above), a constant linear behavior is expected for crystalline materials. For our amorphous structures, a bump is found instead (Fig. 9 (b)), with a clear maximum at about 2.5 meV for the 2.04 g cm^−3^ sample, around 22.3 meV for the 2.80 g cm^−3^ sample, and at 26.5 meV for the 3.48 g cm^−3^ sample. These maxima are comparable to the amorphous lithium borate study by Kojima et al*.*^58^, which vary from 3 to 13 meV depending on the lithium concentration.

## Conclusions

We have studied three structures of a-BN, generated by ab initio Molecular Dynamics within the *undermelt-quench* procedure developed in our group. The densities investigated are 2.04, 2.80 and 3.48 g cm^−3^. When amorphized, most differences in the crystalline structures disappear and the topological arrangements become a “universal” feature which is modulated by the value of the density that may influence the structure and the hybridization of the chemical bond in covalent specimens. The amorphous material then is at best a function of the original density and therefore, this physical property has to be adequately chosen to describe the corresponding final amorphicity.

We calculated the correlation properties of these two samples and compared them with existing ones, both experimentally and simulationally. Our results agree with some and disagree with others. Given the limited amount of existing results for a-BN, it is not surprising that this happens since the statistical fluctuations in such a small universe are large. What is surprising is that there are not more investigations of these properties given the potential applications that this material is claimed to have. More studies are needed along these lines.

The vibrational properties of the structures are determined and found that the a-BN behaves in a similar manner to other amorphous samples; namely, the sharp features of the crystalline disappear and soft features are displayed for the amorphous. The enhancement of the low-frequency modes for the three amorphous samples modifies the thermal and electronic properties of this material; however, a detailed analysis of the electronic structure for the amorphous structures is required in order to obtain a quantitative measure of its effects. Also, soft-phonon modes play an important role in the system, while the optical modes of the crystalline fade away. The vDoS of the three samples are surprisingly similar, perhaps due to the fact that the densities considered are not very different. Nevertheless, the variations in the low-frequency region among different densities could lead to a preference for a specific system to be synthesized having in mind a particular application.

Finally, the calculations of the internal energy and the constant volume specific heat clearly manifest the presence of the soft phonon modes by the appearance of a bump in the plots of *C*_*V*_* /T*^*3*^ and *F(ħω)/(ħω)*^2^, typical behavior of amorphous solids^59^.

## Data Availability

The datasets generated and analyzed during the current study are available from the corresponding author on reasonable request.

## Code availability

Code Correlation can be found in the GitHub repository: https://github.com/Isurwars/Correlation (DOI: 10.5281/zenodo.5514113).
